# *Zingiber officinale* extract maximizes the efficacy of simvastatin as a hypolipidemic drug in obese male rats

**DOI:** 10.1002/fsn3.3889

**Published:** 2023-12-26

**Authors:** Moustafa Salaheldin Abdelhamid, Mohamed Hassan Sherif, Hazem R. Abaza, Lamiaa M. M. El‐Maghraby, Shimaa H. Watad, Ahmed E. Awad

**Affiliations:** ^1^ Biochemistry Department, Faculty of Science Zagazig University Zagazig Egypt; ^2^ Chemistry Department, Faculty of Science Zagazig University Zagazig Egypt; ^3^ Agricultural Biochemistry Department, Faculty of Agriculture Zagazig University Zagazig Egypt

**Keywords:** anti‐obesity, caspase‐3, GLUT‐4, HMG‐CoA reductase, RT‐PCR, *Zingiber officinale*

## Abstract

Obesity became a serious public health problem with enormous socioeconomic implications among the Egyptian population. The present investigation aimed to explore the efficacy of *Zingiber officinale* extract as a hypolipidemic agent combined with the commercially well‐known anti‐obesity drug simvastatin in obese rats. Thirty‐five male Wister rats were randomly divided into five groups as follows: group I received a standard balanced diet for ten weeks; high‐fat diet was orally administered to rats in groups II–V for ten weeks. From the fifth week to the tenth week, group III orally received simvastatin (40 mg/kg B.W.), group IV orally received *Z. officinale* root extract (400 mg/kg B.W.), and group V orally received simvastatin (20 mg/kg B.W.) plus *Z. officinale* extract (200 mg/kg B.W.) separately. Liver and kidney function tests, lipid profiles, serum glucose, insulin, and leptin were determined. Quantitative RT‐PCR analysis of PPAR‐γ, iNOS, HMG‐CoA reductase, and GLUT‐4 genes was carried out. Caspase 3 was estimated in liver and kidney tissues immunohistochemically. Liver and kidney tissues were examined histologically. The administration of *Z. officinale* extract plus simvastatin to high‐fat diet‐fed rats caused a significant reduction in the expression of HMG‐coA reductase and iNOS by 41.81% and 88.05%, respectively, compared to highfat diet (HFD)‐fed rats that received simvastatin only. Otherwise, a significant increase was noticed in the expression of PPAR‐γ and GLUT‐4 by 33.3% and 138.81%, respectively, compared to those that received simvastatin only. Immunohistochemistry emphasized that a combination of *Z. officinale* extract plus simvastatin significantly suppressed caspase 3 in the hepatic tissue of high‐fat diet‐fed rats. Moreover, the best results of lipid profile indices and hormonal indicators were obtained when rats received *Z. officinale* extract plus simvastatin. *Z. officinale* extract enhanced the efficiency of simvastatin as a hypolipidemic drug in obese rats due to the high contents of flavonoid and phenolic ingredients.

## Graphical abstract

*Zingiber officinale* extract has an antiobesity effect by reducing serum total cholesterol and triglycerides and increasing HDL cholesterol levels in hypercholesterolemic rats. *Z. officinale* extract enhanced the efficiency of simvastatin as a hypolipidemic drug in obese rats due to the high contents of flavonoid and phenolic ingredients. The combination of *Z. officinale* extract and simvastatin in HFD‐fed rats caused a significant reduction in the expression of HMG‐coA reductase and iNOS and increased the expression of PPAR‐γ and GLUT‐4 compared to HFD‐fed rats that received SIM only.

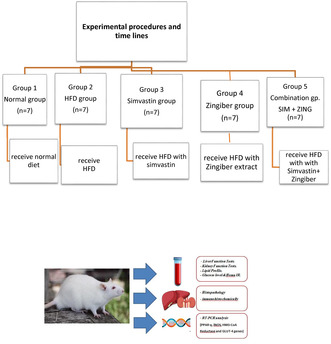

## 1 INTRODUCTION

Obesity is regarded as one of the world's most serious epidemic concerns, posing serious threat to human health. Depending on high‐cholesterol fast meals contented, obesity causes atherogenic dyslipidemia and implicates the pathogenesis of other metabolic syndrome components like hypertension, insulin resistance, and abdominal obesity over time (Grundy, 2016).

Obesity, in particular, has severely raised the prevalence of metabolic disease worldwide (Potabattula et al., 2019). Western diets, which are overwhelming in calories and saturated fat, are associated with obesity (World Health Organization, 2020).

Obesity is described as “abnormal or excess deposit of fats that poses a hazards to healthcare” by the World Health Organization (WHO) (2016). The World Obesity Organization declared obesity to be a chronic, recurrent degenerative disease, dispelling the myth that it is only a risk factor for diseases (Bray et al., 2017).

Insulin‐sensitive tissues are influenced by adipokines. As a result of visceral adiposity dysfunction, changes in adipokine release levels cause changes in body metabolism, insulin sensitivity, and cardiovascular abnormalities (Catalano et al., 2010). As a result, adipokines have been recognized as potential therapeutic targets for obesity and cardiovascular disease treatment. The mainstay of treatment for cardiometabolic problems like atherosclerosis is the reduction of total cholesterol and low‐density lipoprotein‐cholesterol. As a result, cholesterol‐lowering medicines are commonly used; statins are the most well‐known treatment, despite their negative side effects (Yang, Choi, et al., 2011). Alternative medicine based on natural products and plant extracts, in contrast to those medicines, has been utilized on a global scale because of its effectiveness in treating hyperlipidemia and lack of negative side effects (Jo et al., 2014). These extracts have all been investigated as potential anti‐obesity treatments, and several of them have proven to be highly successful (Ahmed et al., 2014; Bais et al., 2014).

Hyperlipidemia is a metabolic and endocrine disorder defined by abnormal lipid metabolism and fat transport difficulties that is currently prevalent all over the world (Liu et al., 2018). The problem of triglycerides and cholesterol is one of the predominant symptoms of hyperlipidemia (Yang, Miyahara, et al., 2011; Wang, et al., 2019). The number of patients with hyperlipidemia has grown in recent years as a result of a high‐cholesterol diet and a sedentary lifestyle, both of which have a negative impact on people's quality of life (Dyer et al., 2014). Currently, medicines utilized to treat hyperlipidemia include simvastatin. However, these treatments have a number of hazards and side effects that make them unsuitable for long‐term or large‐dose use (Boden et al., 2011).

Historically, plants with medicinal properties have been utilized for treating a wide range of illnesses as well as to aid in weight loss and prevent the problems associated with obesity (Bray & Ryan, 2012). Numerous reports of anti‐obesogenic phytonutrients are encouraging (Bahmani et al., 2016; Farhat et al., 2017; Ríos‐Hoyo & Gutiérrez‐Salmeán, 2016; Stohs & Badmaev, 2016).

The ethnopharmacological and ethnobotanical methodologies utilized to determine the therapeutic efficacy of plants offer a strong basis for choosing plants to be further examined in the creation of innovative phytotherapeutic drugs (De Freitas Junior & de Almeida, 2017).

One of the most popular culinary ingredients and spices in the world is ginger, which is made from the rhizomes of the plant Zingiber officinale (Family Zingiberaceae) (Srinivasan, 2017).

Ginger's health benefits include its digestive stimulant action, anti‐inflammatory action, and anticancer effect (Baliga et al., 2012). In rabbits given cholesterol, ginger extract has been noticed to have anti‐hyperlipidemic effects (Elseweidy et al., 2015).

Also, ginger rhizome extracts have been studied to determine their effectiveness in lowering weight gain and expenditure of energy in mouse models fed high‐fat diets (Misawa et al., 2015; Nammi et al., 2009). After taking a supplement containing ginger powder, overweight people in human studies reported having less appetite (Ebrahimzadeh Attari et al., 2018).

Simvastatin is a member of the statin drug class and is used to treat a number of disorders by lowering blood cholesterol (Snarska et al., 2019). Simvastatin had substantial anticoagulant and endothelial cell‐protecting effects (Hu et al., 2009). Simvastatin causes a number of side effects, including gastrointestinal problems, headaches, and rash (Alshekhlee & Katirji, 2007), and some of these impacts can put patients’ lives and health in danger. (Snarska et al., 2018). From this point, the present investigation was designed to evaluate the possibility of using a combination of simvastatin and ginger root extract to reduce the recommended allowed dose of simvastatin and increase its efficacy as a hypolipidemic drug. In order to measure the ability of ginger extract to maximize the efficiency of simvastatin, the expression of hepatic PPARγ, GLUT4 HMG coA reductase and iNOS genes was quantified alongside estimations of blood levels of lipids, insulin, and glucose.

## 2 MATERIALS AND METHODS

### 2.1 Plant material and preparation of the extract

*Zingiber officinale* roots were procured at an area market and identified by Prof. Wafaa M. Amer (Professor of Botany, Faculty of Science, Cairo University). A voucher specimen (voucher no. 6.6.2023) was obtained from the herbarium of the Faculty of Science, Cairo University. The extract of *Zingiber officinale* roots was prepared using the maceration method (Handa et al., 2008; Villegas, 2016). A pressured hot water extraction method was used to extract the ginger powder (modified from Park et al., 2020). For 72 h, 1000 g of powder were soaked in a mixture of 50 cc of ethanol and 50 cc of water. The mixture was then filtered and concentrated at 40°C using a rotating evaporator device. For this study, the extract was pre‐solubilized in distilled water and kept in a tightly closed glass container in the refrigerator between 2 and 8°C.

### 2.2 HPLC analysis of ZING extract

Analyses were performed by HPLC‐(Agilent 1100) is composed of a two LC‐pump, a UV/Vis detector C18 column (125 mm × 4.60 mm, 5 μm particle size). Chromatograms were obtained and analyzed using the Agilent ChemStation phenolic acids. The mobile phase consisted of a binary mixture of methanol/water (50:50 v/v) adjusted to pH 2.8 with phosphoric acid, at an isocratic flow rate of 1.0 mL/min. The following instrument settings were used: nebulizer gas, nitrogen, 40 psi; dry gas, nitrogen, 10 mL/min, 300°C; capillary, −3000 V (+4000 V); end plate offset, −500 V; funnel 1 RF, 200 Vpp; and funnel 2 RF, 200 Vpp. Metabolites were characterized by comparing their mass spectra to the reference literature (Kuntic et al., 2007).

### 2.3 Determination of the total flavonoid content of ZING extract

Estimation of the quantity of total flavonoids was carried out colorimetrically according to the method described by Hoslattmann and Hoslattmann (1982) and Dewanto et al. (2002) as follows: 1.5 mL of deionized water was added to 0.25 mL of ginger root extract, and then 90 μL of 5% sodium nitrite was added. After addition of 180 μL of 10% AlCl_3_, the mixture was allowed to stand for 6 min before mixing with 0.6 mL of 1 M NaOH, and then adding deionized water to adjust the final volume to 3 mL and mixing well. A calibration curve was made by using the standard for total flavonoids (Querestin acid) with absorbance measured at 510 nm and using a blank (measured as mg querestin equivalents (QE) per gram of the sample (QE mg/g)).

### 2.4 Determination of total phenolic contents (TPC) of ZING extract

The concentration of total phenols was measured by a UV spectrophotometer (Jenway‐UV–VIS Spectrophotometer), based on a colorimetric oxidation/reduction reaction, as described by Zheng and Wang (2001).

A calibration curve was created using a standard of gallic acid for TPC using 200 L of 70% ethanolic Zing extract, 1 mL of folin–Ciocalteu reagent (diluted 10 times with distilled water), 8 min of reaction time, 800 L of sodium carbonate (7.5%), and 30 min of standing time. At a wavelength of 765 nm, the absorbance was determined (described as mg of gallic acid equivalent (GAE) per 100 g of dried powder).

### 2.5 Evaluation of antioxidant activity by the DPPH radical scavenging method of ZING extract

By using 1, 1‐diphenyl‐2‐picryl hydrazyl (DPPH), the antioxidant capacity of Zingiber officinale leaves extract was evaluated. Different concentrations (3.9, 7.8, 15.62, 31.25, 62.5, 125, 250, 500, and 1000 g/mL) of ethanolic extract were prepared, then 3 mL of each was mixed separately, with 1 mL of DPPH solution. The mixture was briskly shaken before being left to stand at room temperature for 30 min. Finally, the absorbance was measured using a UV‐VIS Milton Roy spectrophotometer at 517 nm. Ascorbic acid was utilized as the reference. The experiment was carried out in triplicate. The percentage of scavenging potential was calculated according to the following formula:

$$\text{Percent inhibition or DPPH scavenging action}\;\left(\%\right)=A0-A1/\text{A0}\times 100.$$

Where A0 denotes the absorbance of the control reaction and A1 denotes the absorbance when a test or reference sample is present.

### 2.6 Chemicals

Simvastatin was obtained from Global Napi Pharmaceuticals, Egypt. Cholesterol was purchased from Sigma Chemical Company.

### 2.7 Animals

Thirty‐five male Wistar rats weighing 115–120 g were acquired, kept, and stored in the Animal House, Faculty of Medicine, Zagazig University, Egypt. Under standard laboratory conditions, the animals were housed in stainless steel cages and exposed to 12‐h light/dark cycles at 24 ± 2°C and a relative humidity of 60 ± 5%. The experimental protocol was reviewed and approved by the ZU‐IACUC committee (protocol No. ZU‐IACUC/1/F/265/2022).

### 2.8 Experimental design

Rats were divided into five groups, as follows: Group Ι; received a standard balanced diet and ad libitum water for 10 weeks; Group ΙΙ received a high‐fat diet (HFD) consisting of 68% standard chow diet, 30% animal fats, and 2% cholesterol for 10 weeks; Group ΙΙΙ received HFD for 10 weeks and Simvastatin was orally administrated daily (40 mg/kg B.W.) from the fifth week to the tenth week; Group ΙV received HFD for 10 weeks and Zingiber officinale extract was orally administrated daily (400 mg/kg B.W.) daily from the fifth week to the tenth week; and Group V received HFD for 10 weeks, and simvastatin (20 mg/kg B.W.) and Zingiber officinale extract (200 mg/kg B.W.) were orally administrated daily from the fifth week to the tenth week separately (Azza et al., 2013).

### 2.9 Collection of blood and tissue samples

The final body weight of the rats was recorded at the conclusion of the trial period. After fasting overnight, rats were placed down, and blood samples from the retro‐orbital plexus were extracted from each rat while the rodents underwent anesthesia with diethyl ether. To examine hematological parameters, the first blood sample was drawn into di‐potassium EDTA (ethylene diamine tetracetate). The second blood sample was taken in a simple tube to separate the serum for analysis of the lipid profile, leptin, and homa IR, as well as the liver and kidney functions. For the purpose of estimating glucose, the third blood sample was taken from a tube containing sodium fluoride. For histopathological analysis, kidneys, livers, and pancreas were taken apart and fixed in 10% formalin.

### 2.10 Histopathology

Liver and kidney tissue specimens were collected from different groups and fixed in 10% buffered neutral formalin. Paraffin blocks were prepared for examination under a light microscope (Survarna et al., 2013).

### 2.11 Immunohistochemistry

Further sections on positively charged coated slides were used for the IHC technique using monoclonal and polyclonal antibodies marking the target element. Cytochrome‐c primary antibody was used for estimation of mitochondrial membrane integrity, and caspase‐3 primary antibody was used as an indicator for apoptosis induction, according to Atef et al. (2018).

### 2.12 RT‐PCR analysis

#### 2.12.1 RNA extraction

Using the QIAamp RNeasy Mini kit (Qiagen, Germany, GmbH), RNA was extracted from tissue samples by adding 200 L of the sample to 600 L of RLT buffer, which contained 10 L of ‐mercaptoethanol per 1 mL, and incubating at room temperature for 10 min. Following the instructions in the Purification of Total RNA technique of the QIAamp RNeasy Mini kit (Qiagen, Germany, GmbH), one volume of 70% ethanol was added to the cleared lysate. N.B. To eliminate the remaining DNA, DNase digestion was performed on the column.

#### 2.12.2 Oligonucleotide primers

The primers were provided by Metabion (Germany), and are listed in Table 1.

**TABLE 1:** Primers.

Target gene	Primers sequences	Reference
B‐Actin	CCTGCTTGCTGATCCACA	Patel et al. (2013)
	CTGACCGAGCGTGGCTAG	
GLUT4	GCCTTCTTTGAGATTGGTCC	Patel et al. (2013)
	CTGCTGTTTCCTTCATCCTG	
PPAR γ	GGATTCATGACCAGGGAGTTCCTC	Patel et al. (2013)
	GCGGTCTCCACTGAGAATAATGAC	
HMG‐CoA reductase	CAGGATGCAGCACAGAATGT	Wu et al. (2013)
	CTTTGCATGCTCCTTGAACA	
iNOs	CACCACCCTCCTTGTTCAAC	Sobajima et al. (2005)
	CAATCCACAACTCGCTCCAA	

#### 2.12.3 cDNA synthesis

A 25 μL reaction was used to test the primers, which included 10 μL of the 2× HERA SYBR® Green RT‐qPCR Master Mix (Willowfort, UK), 1 μL of RTEnzyme Mix (20×), 0.5 μL of each primer at a concentration of 20 pmol, 5 μL of water, and 3 μL of RNA template. A real‐time PCR step one machine was used to carry out the reaction.

The amplification curves and Ct values were determined by the step one software. The Ct of each sample was compared with that of the positive control group using the “Ct” method published by Yuan et al. (2006)) to estimate the variation in gene expression on the RNA of the various samples using the following ratio (2^−ΔΔCt^). The amplification was performed under the conditions listed in Table 2.

**TABLE 2:** The conditions for amplification.

Reverse transcription	Primary denaturation	Amplification (40 cycles)		
		Secondary denaturation	Annealing (Optic's on)	Extension
65°C 60 min	95°C 15 min	95°C 15 s	60°C 1 min	72°C 30 s

### 2.13 Statistical analysis

Data were expressed in tables and figures as mean (*M*) ± standard deviation (SD). The experimental results were analyzed statistically using SPSS version 20.0. A one‐way ANOVA was carried out to test the significant differences among all experimental groups. Moreover, a Student *t*‐test was performed to determine the significant difference between the control group and the rest of the experimental groups, and all differences were considered statistically significant when *p* ≤ .05. The graphs of gene expression were created using GraphPad Prism software (version 8).

## 3 RESULTS

### 3.1 The antioxidant capacity of ZING extract

As shown in Table 3, the IC_50_ of ZING extract was recorded at 11.87 μg/mL.

**TABLE 3:** The in vitro antioxidant scavenging capacity of ZING extract.

Sample (Zing.) (Conc. μg/mL)	OD mean	DPPH scavenging%
1000	0.096	92.8
500	0.119	91.1
250	0.204	84.8
125	0.315	76.5
62.5	0.425	68.3
31.25	0.511	61.9
15.625	0.641	52.2
7.8125	0.734	45.3
3.9	0.857	36.1
1.95	0.924	31.1

### 3.2 Total content of flavonoids and phenolic compounds in ZING extract

Our results showed that *ZING* extract *contains* flavonoid 15.52 mg QU/g sample and phenolic compounds 110.23 mg GAE/g DE.

### 3.3 HPLC chromatogram

**FIGURE 1:**
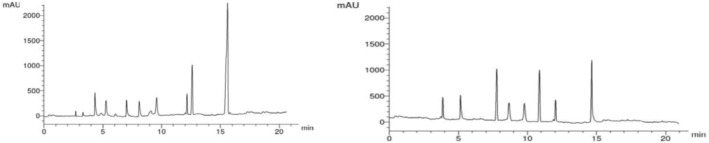
Total flavonoids and total phenolic contents of ZING.

As shown in Figure 1 and Table 4, the phenolic compound contents (μg/mg DW) in Table 4 revealed that ZING contains many phenolic compounds such as gallic acid, catechin, caffeic acid, syringenic acid, ellagic, salicylic acid, protocatechulic, naringin, rutin, kampferol, luteolin, hisperdin, chrysoeriol, cinnamic, and 7‐OH flavone and high amount for protocatchuic (35.16 μg/mL DW), ellagic (17.36 μg/mL DW), and chrysoeriol (15.36 μg/mL DW), respectively. Also, less amount of Luteolin 2.36 μg/mL DW) is found.

**TABLE 4:** Total flavonoids and total phenolic contents of *ZING.*

RT#	Compound	Conc (μg/mL)
4.5	Catechin	6.33
5.1	Syringenic	4.15
7.0	Cinnamic	5.36
8.0	Caffeic	5.47
9.8	Gallic	5.06
12.0	Salicylic	4.89
12.8	Ellagic	17.36
15.6	Protocatechulic	35.16
4.0	Naringin	5.22
5.0	Rutin	4.69
8.0	Kampferol	11.14
8.7	Luteolin	2.36
9.8	Hisperdin	3.05
11.0	7‐OH flavone	10.14
15.0	Chrysoeriol	15.36

The extract of *Zingiber officinale* contained flavonoids and phenolics that are related to its usage in the treatment of various disorders in traditional systems of medicine.

### 3.4 The effect of ZING extract and SIM on body weight

The percent change in body weight of rats among test groups from the initial weight is recorded in Table 5. It was noticed that the best effect on body weight was observed when HFD‐fed rats received a combination of SIM and ZING.

**TABLE 5:** The effect of Zingiber officinale extract (ZING), simvastatin (SIM), and their combination on body weight gain (g) in rats fed a high‐fat diet (HFD) for 6 weeks. All values are means ± SD (*n* = 7).

Weeks	Groups				
	Normal control	HFD	HFD + SIM	HFD + ZING	HFD + SIM + ZING
1st week	150 ± 5.3	240 ± 6.4	240 ± 5.6	240 ± 5.4	240 ± 5.1
2nd week	158 ± 3.9	256 ± 4.2	247 ± 3.2	253 ± 4.2	246 ± 3.2
3rd week	167 ± 4.7	274 ± 7.1	252 ± 4.7	260 ± 4.7	253 ± 3.7
4th week	182 ± 3.9	295 ± 5.3	259 ± 3.7	270 ± 5.2	260 ± 4.5
5th week	194 ± 4.5	320 ± 6.8	267 ± 4.9	278 ± 7.3	266 ± 4.5
6th week	200 ± 4.9	345 ± 5.7	272 ± 3.2	287 ± 3.5	271 ± 2.9
% of change from initial weight	33.3	43.75	13.3	19.58	12.91

### 3.5 The effect of ZING extract and SIM on liver and kidney function parameters

As shown in Tables 6 and 7, it was noted that serum levels of liver enzymes (ALT and AST) in HFD‐fed rats receiving ZING showed a significant effect on liver enzymes (*p* ≤ .001) by 32.39% and 16.21%, respectively compared to HFD group. Similarly, When administrated *ZING* in a combination with SIM showed the best effect as it significantly decreased ALT by 39.44% and AST by 20.27% (*p* ≤ .001) when compared to HFD group.

**TABLE 6:** Variations of liver and kidney function tests indicators among the experimental group. All values are means ± SD (*n* = 7).

Group/parameter	Liver function				Kidney function		
	Alt (U/L)	AST (U/L)	Alp. (U/L)	ALB. (g/dL)	Creat. (mg/dL)	Uric acid (mg/dL)	BUN (mg/dL)
Neg. Control	34 ± 2.7	105 ± 4.3	237 ± 4.7	4.9 ± 0.4	0.54 ± 0.07	1.9 ± 0.14	14 ± 2.5
HFD	71 ± 2.36	148 ± 2.8	680 ± 5.17	3.85 ± 0.31	0.82 ± 0.036	2.78 ± 0.04	33.8 ± 1.14
HFD + SIM	45.6*** ± 2.58	121*** ± 3.1	273*** ± 2.73	4.25* ± 0.18	0.68*** ± 0.04	2.16*** ± 0.04	24.4*** ± 1.68
HFD + ZING	48*** ± 2.36	124*** ± 3.1	305*** ± 3.93	4.58*** ± 0.24	0.71** ± 0.06	2.5*** ± 0.04	29.3*** ± 0.75
HFD + SIM + ZING	43*** ± 2.6	118*** ± 2.42	270*** ± 3.26	4.6*** ± 0.27	0.67*** ± 0.03	2.12*** ± 0.03	23.5*** ± 0.77

*Note*: Results were expressed as mean ± SD. Superscript stars indicate significant differences between test groups and control group, where (*) indicates *p* < .05 mildly significant, (**) *p* < .01 significant, (***) *p* < .001 highly significant, and NS, non‐significant.

**TABLE 7:** Variations of the percent of change of biochemical parameters among the experimental groups compared to HFD control group.

Groups	Parameters						
	Liver functions tests				Kidney functions tests		
	ALT % change (decrease)	AST % change (decrease)	ALP % change (decrease)	ALB. % change (increase)	Creat. % change (decrease)	UA % change (decrease)	BUN % change (decrease)
HFD + SIM	35.77	18.24	59.85	10.39	17.07	22.3	27.81
HFD + ZING	32.39	16.21	55.14	18.96	13.41	10.07	13.31
HFD + SIM + ZING	39.44	20.27	60.29	19.48	18.29	23.74	30.47

*Note*: % change was calculated compared to HFD control group.

Our results showed that the best effect of *ZING* when used in combination with SIM in liver function test. The activity of ALP significantly decreased (*p* ≤ .001) by 55.14 % and ALB significantly increased (*p* ≤ .001) by 18.96 6% in HFD‐feed rats received ZING compare to HFD‐feed rats group but when used in combination with SIM showed best effect, where ALP significantly decreased (*p* ≤ .001) by 60.29% and ALB significantly increased (*p* ≤ .001) by 19.48 % compared to HFD group.

The results of kidney function tests demonstrated that *ZING* enhanced kidney functions, where it significantly decreased creatinine by 13.41%, UA by 10.07 % and BUN by 13.31 % when adminstrated individually to HFD‐feed rats (compared to HFD group but this ameliorative activity became more potent when ZING was adminstrated in combination with SIM that evidenced by significantly decreasing creatinine by 18.29 %, UA by 23.74 % and BUN by 30.47 % compared to HFD group.

The best effect for liver and kidney function tests was obtained at the administration of a combination of *ZING* plus SIM.

### 3.6 The effect of ZING extract and SIM on glucose, insulin, HOMA IR, and leptin levels

As shown in Tables 8 and 9, it was observed that *ZING* extract decreased glucose level significantly (*p* ≤ .05) by 21.66 % when administrated individually to HFD‐fed rats compared to HFD group, similarly HOMA IR mildly significantly decreased (*p* ≤ .05) by 21.87% and leptin highly significantly decreased (*p* ≤ .001) by 43.15% in HFD‐fed rats received *ZING* individually compared to the HFD‐fed rat group, and this effect increases by using ZING in combination with SIM, which showed the best results as significantly decreasing glucose (*p* ≤ .01) by 33.33 %, highly significantly decreasing HOMA IR (*p* ≤ .001) by 43.75 %, and highly significantly decreasing leptin (*p* ≤ .001) by 49.58% when compared to HFD group.

**TABLE 8:** Variations in blood glucose level and hormonal indicators among the experimental groups. All values are presented as mean ± SD (*n* = 7).

Group/parameter	Glucose (mg/dL)	Insulin (nIU/mL)	HOMA IR	Leptin (ng/mL)
Neg. control	112 ± 3.5	0.13 ± 0.02	0.12 ± 0.07	3.1 ± 0.7
HFD	180 ± 30.1	0.37 ± 0.03	0.32 ± 0.04	7.3 ± 0.39
HFD + SIM	124*** ± 3.9	0.16*** ± 0.03	0.19*** ± 0.021	3.83 *** ± 0.43
HFD + ZING	141* ± 5.26	0.19*** ± 0.021	0.25** ± 0.034	4.15*** ± 0.31
HFD + SIM + ZING	120** ± 2.6	0.15*** ± 0.026	0.18*** ± 0.023	3.68*** ± 0.33

*Note*: Results were expressed as mean ± SD. Superscript stars indicate significant differences between test groups and control group, where (*) indicates *p* < .05 mildly significant, (**) *p* < .01 significant, (***) *p* < .001 highly significant, and NS, non‐significant.

**TABLE 9:** Variations of the percent of change among the experimental groups compared to HFD control group.

Group/parameter	Glucose % change (decrease)	Insulin % change (decrease)	HOMA IR % change (decrease)	Leptin % change (decrease)
HFD + SIM	31.11	56.75	40.62	47.53
HFD + ZING	21.66	48.64	21.87	43.15
HFD + SIM + ZING	33.33	59.45	43.75	49.58

*Note*: % change was calculated compared to HFD control group.

The best effects for glucose, insulin, HOMA IR, and leptin were obtained from the administration of a combination of ZING and SIM.

### 3.7 The effect of ZING extract and SIM on the lipid profile test

#### 3.7.1 The percentage of change was calculated compared to the HFD control group

According to the results in Tables 10 and 11, it was noted that *ZING* in a combination with SIM has the best effect on lipid profile, as when used individually, it highly significantly decreased cholesterol by 49.52 % and TAG by 27.1% (*p* ≤ .001) compared to the HFD  group, but the best effect is shown when *ZING* was combined with SIM, which highly significantly deceased cholesterol by 53.96% and TAG by 41.17 % (*p* ≤ .001) compared to HFD group.

**TABLE 10:** Variations of Lipid Profile Indices among the Experimental Groups. All values are presented as mean ± SD (*n* = 7).

Group/parameter	Cholest. (mg/dL)	TAG (mg/dL)	HDL (mg/dL)	LDL (mg/dL)	VLDL (mg/dL)
Neg. Control	107 ± 4.8	64 ± 3.7	69 ± 2.1	25.2 ± 2.4	12.8 ± 3.2
HFD	315 ± 4.5	128 ± 4.27	37.16 ± 3.06	251.8 ± 6.8	26 ± 2.36
HFD + SIM	150*** ± 4.3	80*** ± 3.34	50*** ± 2.58	73.6*** ± 2.16	17.16*** ± 2.85
HFD + ZING	159*** ± 4.14	93.3*** ± 2.65	44.16*** ± 2.6	93.83*** ± 3.06	19.6*** ± 2.16
HFD + SIM + ZING	145*** ± 4.32	75.3*** ± 3.14	54*** ± 2.6	68.16*** ± 3.25	14.5*** ± 1.87

*Note*: Result were expressed as mean ± SD. Superscript stars indicate significant differences between test groups and control group, where (*) indicates *p* < .05 mildly significant, (**) *p* < .01 significant, (***) *p* < .001 highly significant, and NS, non‐significant.

**TABLE 11:** Variations of the percent of change among the experimental groups compared to HFD control group.

Group/parameter	Cholest. % change (decrease)	TAG % change (decrease)	HDL % change (increase)	LDL % change (decrease)	VLDL % change (decrease)
HFD + SIM	52.38	37.5	34.55	70.77	34.0
HFD + ZING	49.52	27.1	18.83	62.73	24.61
HFD + SIM + ZING	53.96	41.17	45.31	72.93	44.23

The best effect for lipid profile tests obtained in the rats group was a combination of ZING and SIM.

### 3.8 The effect of ZING and SIM on the histopathology of the liver

**FIGURE 2:**
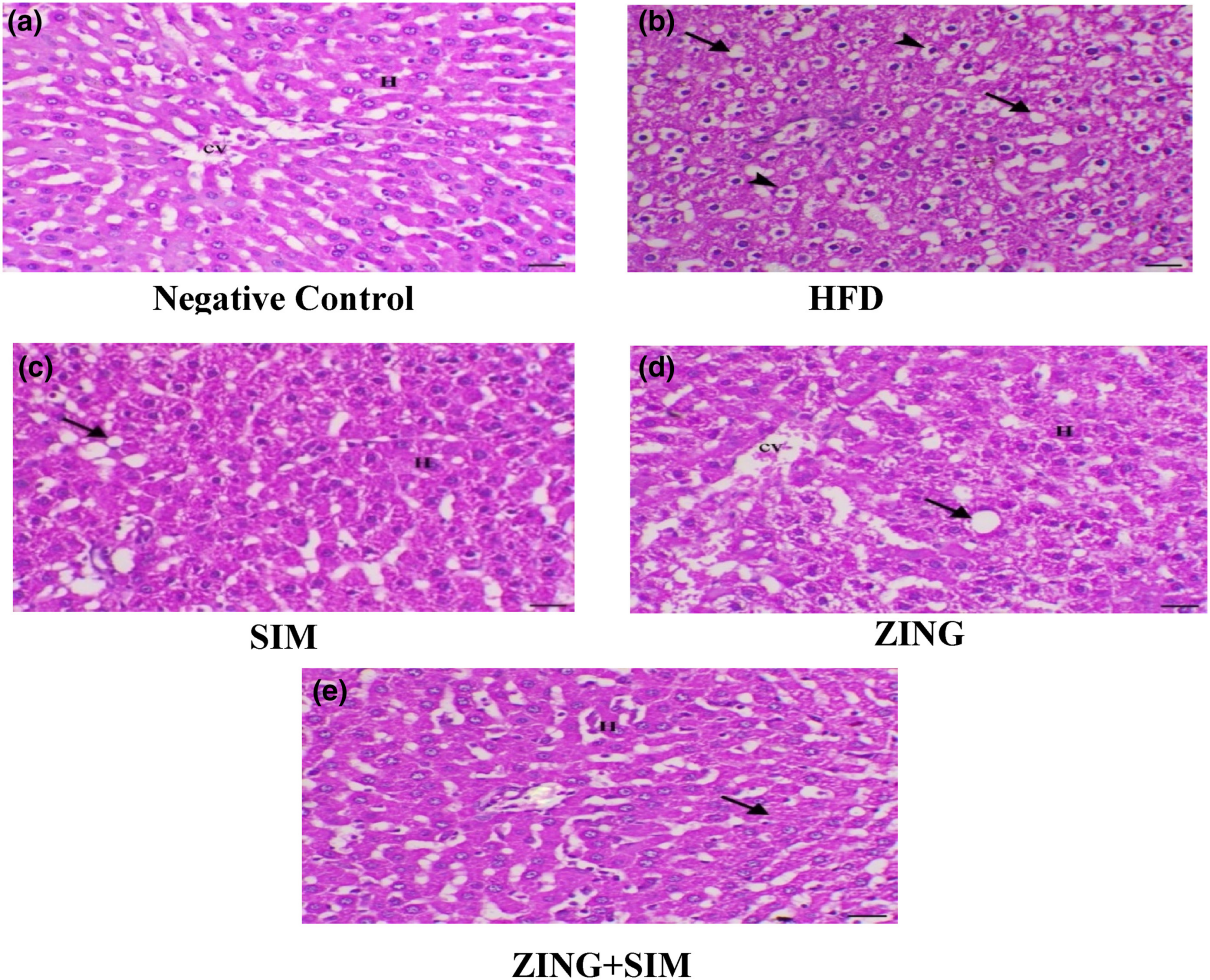
The effect of ZING and SIM on liver histopathology. (a) Liver of G1 animal showing normal hepatocytes around the central vein (H indicates hepatocytes and cv indicates central vein), H&E, ×200, bar = 50 μm, (b) liver of G2 animal showing diffuse marked hepatic vacuolation associated with both fatty changes (arrows) and glycogen accumulation within hepatocytes (arrowheads), H&E, ×200, bar = 50 μm, (c) liver of G3 animal showing decrease fatty changes with granular vacuolar degeneration within hepatocytes (arrow indicates fat vacuole and H indicates hepatocytes), H&E, ×200, bar = 50 μm, (d) liver of G4 animal showing decreased fatty changes with moderate degree of granular vacuolar degeneration (arrow indicates fat vacuole and H indicates hepatocytes), H&E, ×200, bar = 50 μm, (e) liver of G5 animal showing marked decrease in fat accumulation and granular changes within hepatocytes (arrows), H&E, ×200, bar = 50 μm.

As shown in Figure 2, it was noticed that the administration of ZING extract combined with SIM led to improvements in the histopathological criteria of liver tissue, as evidenced by the disappearance of fat accumulation and the reforming of the remnants of vacuolar degeneration within hepatocytes compared to rats receiving a high‐fat diet without treatment.

### 3.9 The effect of ZING and SIM on the histopathology of kidney

**FIGURE 3:**
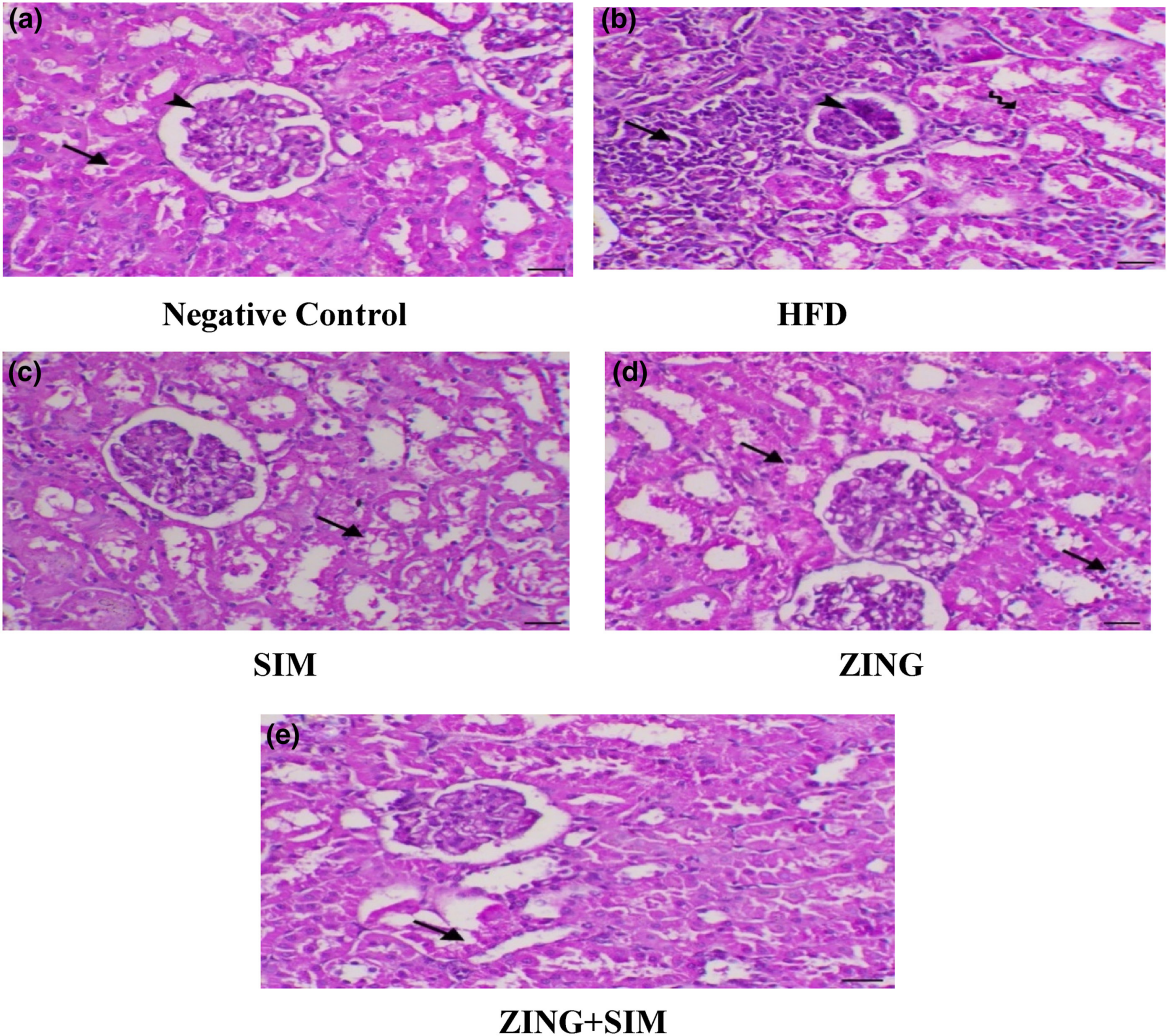
The effect of ZING and SIM on kidney histopathology. (a) Kidney of a G1 animal showing normal renal glomeruli and tubules (arrowhead and arrow, respectively), H&E, ×200, bar = 50 μm, (b) Kidney of a G2 animal showing features of interstitial nephritis associated with glomerular sclerosis (arrowheads), tubular degeneration (tailed‐arrow), and interstitial inflammatory cell infiltration, mostly lymphocytes and macrophages (arrow), H&E, ×200, bar = 50 μm, (c) Kidney of a G3 animal showing marked decrease in lesions with still noticeable vacuolar tubular degeneration (arrow), H&E, ×200, bar = 50 μm, (d) Kidney of a G4 animal showing a moderate degree of tubular degeneration (arrows indicate vacuolation of tubular epithelium), H&E, ×200, bar = 50 μm, (e) Kidney of a G5 animal showing a marked decrease in tubular degeneration (arrow), H&E, ×200, bar = 50 μm.

As shown in Figure 3, HFD‐fed rats that received ZING extract exhibited an inflammatory injury in renal tissue to a lesser extent than the HFD control group, despite the fact that the best result was obtained when ZING extract was administered in combination with SIM, indicating a decrease in inflammatory lesions and vacuolar tubular degeneration in the kidney tissues of that group compared to the control group.

### 3.10 The effect of ZING and SIM on the hepatic expression of caspase‐3 in all experimental groups (*n* = 7) (Figure 4)

**FIGURE 4:**
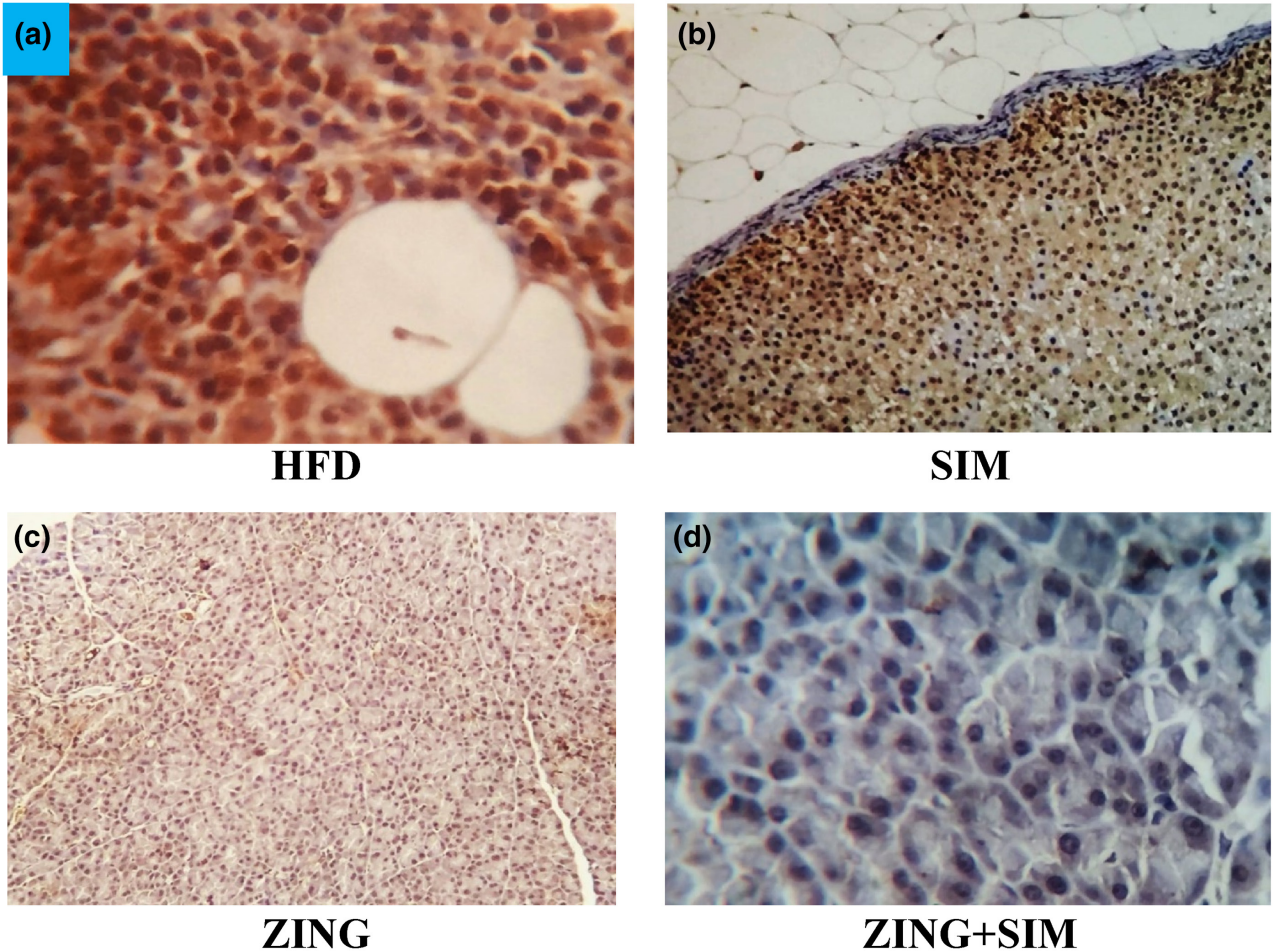
Variation of the hepatic expression of caspase‐3 among the experimental groups. (a) Photomicrograph of a liver section from a rat of group **(HFD)** showing perivascular hepatic cells with *intense positive* cytoplasmic reactivity for caspase ‐3(peroxidase ×400), (b) Photomicrograph of a liver section from a rat of group **(SIM)** showing diffuse subcapsular hepatic cells with *moderate positive* cytoplasmic reactivity for caspase ‐3(peroxidase ×200), (c) Photomicrograph of a liver section from a rat of group **(ZING)** showing hepatic cells within hepatic parenchyma with *weak positive* cytoplasmic reactivity for caspase ‐3 ( peroxidase ×200), (d) Photomicrograph of a liver section from a rat of group **(SIM+ZING)** showing hepatic cells in the hepatic parenchyma with *negative* cytoplasmic reactivity for caspase ‐3 (peroxidase × 400).

#### 3.10.1 The effect of ZING extract and SIM on the expression of hepatic HMG‐coA reductase, iNOS, GLUT4, and PPAR‐γ genes

**FIGURE 5:**
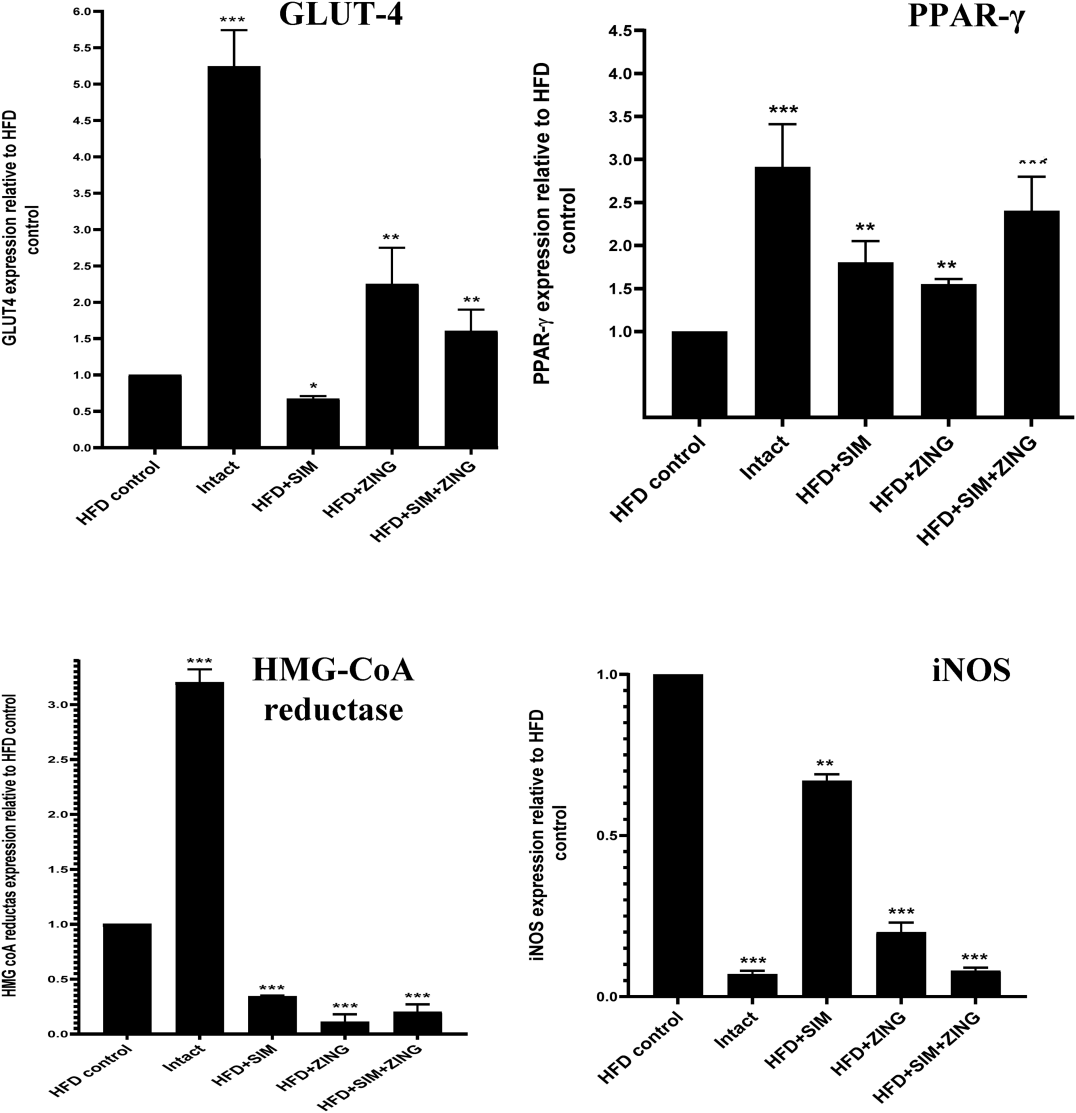
The effect of ZING and SIM on the expression of HMG‐CoA reductase, iNOS, GLUT4, and PPAR‐γ genes in all experimental groups. The results were expressed as mean ± SD. Superscript stars indicate significant differences between test groups and the HFD control group, where (*) indicates *p* < .05 mildly significant, (**) *p* < .01 significant, (***) *p* < .001 highly significant, and NS, non‐significant.

As shown in Figure 5, it was noticed that the administration of a combination of ZING plus SIM to HFD‐fed rats caused an increase in the expression of the GLUT‐4 gene by 138.81% compared to a group of rats receiving SIM only (*p* < .001). Likewise, the expression of the PPAR‐γ gene was increased by 33.3% in HFD‐fed rats receiving a combination of ZING plus SIM compared to a group receiving SIM only (*p* < .01). On the contrary, a decrease of 41.81% and 88.05% in the expression of HMG‐CoA reductase and iNOS genes, respectively, was noticed in HFD‐fed rats receiving a combination of ZING plus SIM compared to a group receiving SIM only (*p* < .001).

## 4 DISCUSSION

Obesity is considered an avoidable cause of death globally (Mehrzad, 2020), and Egypt has an advanced ranking in the prevalence of obesity worldwide (ProCon.org. Global Obesity Levels, 2020). Over the years, numerous drugs have been utilized to treat obesity, such as Orlistat, Sibutramine, Simvastatin, phentermine‐topiramate, and liraglutide, but the majority of these anti‐obesity medications that were licensed and promoted have now been discontinued because of substantial side effects. The addition of medicinal herb extract to a well‐known hypolipidemic pharmaceutic may be beneficial to improve its efficacy and reduce its recommended therapeutic dose.

Zingiber officinale (ZO) is one of the most well‐known medicinal plants, characterized by its powerful biopotentials. The antioxidant potential of ZO is attributed to the presence of many bioactive constituents in its extract. In the present investigation, HPLC chromatograms showed that ZO extract contains protocatchuic (35.16 μg/mL DW), ellagic (17.36 μg/mL DW), and chrysoeriol (15.36 μg/mL DW) as major components that have biological properties and pharmacological activities such as antioxidant, antibacterial, anticancer, antiulcer, antidiabetic, antiaging, antifibrotic, antiviral, anti‐inflammatory, analgesic, antiatherosclerotic, cardiac, hepatoprotective, neurological, and nephroprotective (Kakkar & Bais, 2014).

Due to the presence of protocatchuic as a major component in ZING extract, which showed its activity in lipid profiles that decreased serum TC, serum TG, LDL‐c, and VLDL levels when compared to the control group, a result reported by Liang et al. (2020) assumed that protocatchuic played an effective role as a cardio‐protectant similar to that of simvastatin.

Also, the presence of ellagic acid in ZING extract is considered to be efficient at preventing oxidative harm from cells (Hussein & Khalifa, 2014) and enhancing the antioxidant defense system's activity (Seeram et al., 2005); as it has strong antioxidant potential (Karimi et al., 2011; Sarker & Oba, 2019), it enhanced the serum lipid profile in mice fed a high‐fat diet (Yoshimura et al., 2013) In rats given a high‐fat, fructose‐containing diet, ellagic acid supplementation reduced serum triacylglycerol and cholesterol levels (Amin & Arbid, 2017) and decreased LDL and increased HDL in rats fed a high‐fat diet (Nankar, Doble, et al., 2017).

Concerning the hypolipidemic activity of ZO, the findings of the present investigation concluded that serum cholesterol and serum TG in HFD‐fed rats receiving ZING+SIM decreased by 3.0% and 5.8%, respectively, compared to HFD‐fed rats receiving SIM alone, and HDL level in HFD‐fed rats receiving ZING + SIM increased by 8.0% compared to HFD‐fed rats receiving SIM alone; a similar result was reported in a previous study conducted by Ullagaddi et al. (2020) that reported ZING extract increased HDL level (*p* < .001) and decreased TC, TG (*p* < .01), and VLDL (*p* < .01). Our result showed a better effect than the results reported by Akram Irannejad et al. (2020)) and (Nazish et al., 2016) who used Zing extract and showed increased HDL cholesterol (*p* < .05), but the level of LDL cholesterol did not show a significant change in Zing extract groups compared with the HFD group, and also previous studies by Wang et al. (2019) and Rahimlou et al. (2019) showed that Zing extract significantly lowers serum levels of both LDL and total cholesterol. In agreement with our results, Bin‐Meferij et al. (2017) concluded that the administration of Zing extract effectively decreased obesity. Along with the HFD, serum total cholesterol (TC), LDL, and triglycerides were significantly lowered and HDL levels increased. Additionally, our outcomes were compatible with the report from Borekar et al. (2020), which emphasized that the best effect was achieved when Zingiber extract was used at 400 mg/kg body weight.

In respect to the hepatoprotective effect of Zing, our outcomes showed that HFD‐fed rats receiving ZING+SIM exhibited a significant decrease in ALT, AST, and ALP activities, as well as a decrease in creatinine, BUN, and uric acid by 5.7%, 2.47%, 1.09%, 1.47%, 3.68%, and 1.85%, respectively, and a significant increase in serum albumin by 8.25% compared to HFD‐fed rats receiving SIM alone. The report of the World Health Organization (2016) concluded that the Zing extract protects the rat liver from damage brought on by acetaminophen intoxication. Simvastatin, the reference medicine, on the other hand, resulted in a degree of liver damage that was linked to a reduction in ALT activity, as mentioned by Yang, Choi, et al. (2011). The results of creatinine and BUN, which are extremely similar to normal control values, show that Zingiber officinale extract combined with simvastatin appears to be safe for kidneys and keep away from nephrotoxicity (Dooley et al., 2018). In accordance with our results, a previous report conducted by Irannejad et al. (2020) revealed that, in comparison to the HFD group, Zing extract significantly lowers serum levels of AST and ALT (*p* < .05).

Because of the entry of high glucose levels into the mitochondria in hyperglycemia, the beta cells are damaged. With apparent hyperglycemia and a higher HOMA‐IR, the obese group acquired insulin resistance (Kameshwaran et al., 2013). In the present study, our results emphasized that using the Zingiber extract in combination with simvastatin enhanced glucose level, insulin level, HOMA IR, and leptin by 3.2%, 6.25%, 5.26%, and 3.9%, respectively, compared to HFD‐fed rats receiving SIM alone. These findings were consistent with previous reports revealing that Zing extract reduced body weight gain, obesity, and serum leptin content (Nazish et al., 2016). This result was better than that reported by Nazish et al. (2016)), which showed a non‐significant decrease in insulin and leptin compared to the HFD group.

In obesity, several extrinsic and intrinsic signaling mechanisms can cause caspase‐dependent apoptotic cell death. Obese human adipose tissue has been discovered to have elevated amounts of active caspase‐3, ‐7, and ‐9 protein and caspase‐3/7 activity as well as decreased phosphorylation of anti‐apoptotic Bcl2 protein (Tinahones et al., 2013). In the present study, our results emphasized that Zingiber extract in combination with simvastatin showed a weakly reaction compared to the HFD group in sections of the liver and kidney.

Regarding RT‐PCR analysis of PPARγ, the administration of ZING+SIM to HFD‐fed rats upregulated its expression by 25.2% compared to a group that received SIM only. In the case of hypolipidemia, as a reinforcement lipogenic mechanism to sterol receptor element‐binding protein 1c (SREBP‐1c) activation, obesity reduced liver PPAR expression (Pettinelli & Videla, 2011). Adipocyte differentiation and fat accumulation were inhibited by *Zingiber officinale* extract's impact on the PPAR pathway in liver and adipose tissue. Our results, in agreement with previous studies, concluded that the administration of Zingiber extract increased mRNA expression of PPAR‐α compared to the HFD rat group. A similar result was reported in a previous study conducted by Seok Hee Seo et al. (2021), who concluded that ZING extract upregulated the expression of hepatic PPARγ compared to the HFD group.

Concerning RT‐PCR analysis of HMG‐Co‐A reductase, a combination of ZING and SIM rats feeding HFD downregulated its expression by 35.61% compared to a group receiving SIM alone.

When compared to the HFD group, the total cholesterol level substantially decreased in the ZING extract groups. According to reports, Zing extract may enhance cholesterol's conversion to bile acids, inhibit cholesterol production, and boost fecal excretion (Verma et al., 2004). The findings of our present study are in agreement with previous reports that revealed that the administration of an ethanolic extract of Zingiber officinale caused a decrease in serum cholesterol levels in HFD‐fed rats (Ajayi, 2011; Al‐Kishu et al., 2013). The hypocholesterolmic effect of Zingiber officinale downregulated HMG‐CoA reductase activity (Srinivas et al., 2010).

Insulin‐mediated glucose absorption in adipose tissue and skeletal muscle is carried out by the glucose transporter protein GLUT4, which is crucial for maintaining glycemic homeostasis. Based on RT‐PCR analysis in the present study, ZING+SIM upregulated the expression of GLUT4 by 72.89% compared to HFD receiving SIM alone.

The expression of GLUT4 in adipose tissue and skeletal muscle is expected to be downregulated in hyperglycemia, resulting in insulin resistance (Hu et al., 2014). Further, the combination of Zingiber officinale with Simvistatin showed enhanced GLUT‐4 translocation to increase glycogen production in skeletal muscle (Samad et al., 2017). These *Zingiber officinale* properties could be the explanation for the rats’ improved glucose metabolism, so based on our outcomes in the present study, our results emphasized that the Zingiber extract in combination with simvastatin increased GLUT4 compared to the SIM group.

iNOS, a crucial NO enzyme that increase NEFA, can promote the generation of inflammatory mediators and oxidative stress when there is a high dietary fat intake (Ghosh et al., 2017). Increased NO levels may promote lipolysis and fatty acid oxidation in fat cells, resulting in the inflammatory cascade that leads to insulin resistance and endothelial dysfunction (Hong et al., 2015; Jobgen et al., 2006). Due to a lack of NO substrate that decreases NO bioavailability by activating NADPH oxidase activity, upregulation of endothelium iNOS in obese rats may be related to O_2_ production (Lobato et al., 2012). Our result showed that Zingiber extract downregulated iNOS compared to the HFD group, and the combination of the extract with simvastatin showed a downregulation of 32.12% compared with the group treated with SIM group.

## 5 CONCLUSION

In accordance with our findings, ZING extract maximized the efficacy of SIM as a hypolipidemic drug in obese rats, and this effect is evidenced by upregulating of GLUT‐4 and PPAR‐γ as well as downregulating of iNOS and HMG‐coA reductase. Furthermore, the addition of ZING extract to SIM led to the best results in lipid profile indices and hormonal indicators. An amelioration of liver and kidney functions was noticed in HFD‐fed rats that received ZING+SIM better than those that received SIM only.

## AUTHOR CONTRIBUTIONS

**Moustafa Salaheldin Abdelhamid:** Conceptualization (equal); formal analysis (equal); investigation (equal); methodology (equal); project administration (equal); resources (equal); software (equal); supervision (equal); validation (equal); visualization (equal); writing – original draft (equal); writing – review and editing (equal). **Mohamed Hassan Sherif:** Supervision (equal); validation (equal). **Hazem R. Abaza:** Conceptualization (equal); data curation (equal); formal analysis (equal); investigation (equal); methodology (equal); writing – original draft (equal); writing – review and editing (equal). **Lamiaa M. M. El‐Maghraby:** Data curation (equal); formal analysis (equal); methodology (equal). **Shimaa H. Watad:** Data curation (equal); formal analysis (equal); methodology (equal); project administration (equal). **Ahmed E. Awad:** Data curation (equal); investigation (equal); methodology (equal); resources (equal); software (equal); validation (equal); visualization (equal); writing – original draft (equal); writing – review and editing (equal).

## CONFLICT OF INTEREST STATEMENT

The authors have no potential conflicts of interest.

## ETHICS STATEMENT

This protocol has been reviewed and approved by ZU‐IACUC committee (approval number: ZU‐IACUC/1/F/265/2022; duration of approval: 26/10/2022 to 26/10/2025).

## Data Availability

Data will be made available from the corresponding author upon request.
